# Anomaly Detection Framework for Wearables Data: A Perspective Review on Data Concepts, Data Analysis Algorithms and Prospects

**DOI:** 10.3390/s22030756

**Published:** 2022-01-19

**Authors:** Jithin S. Sunny, C. Pawan K. Patro, Khushi Karnani, Sandeep C. Pingle, Feng Lin, Misa Anekoji, Lawrence D. Jones, Santosh Kesari, Shashaanka Ashili

**Affiliations:** 1 Rhenix Lifesciences, Hyderabad 500038, India; jithin@rhenix.org (J.S.S.); khushi@rhenix.org (K.K.); 2 CureScience, San Diego, CA 92121, USA; spingle@curescience.org (S.C.P.); flin@curescience.org (F.L.); manekoji@curescience.org (M.A.); ljones@curescience.org (L.D.J.); shashi@curescience.org (S.A.); 3 Pacific Neuroscience Institute, Providence Saint John’s Health Center, Santa Monica, CA 90404, USA; santoshkesari@gmail.com; 4 Department of Translational Neurosciences, Saint John’s Cancer Institute at Providence Saint John’s Health Center, Santa Monica, CA 90404, USA

**Keywords:** anomaly detection, heart rate, wearables, missing data, machine learning

## Abstract

Wearable devices use sensors to evaluate physiological parameters, such as the heart rate, pulse rate, number of steps taken, body fat and diet. The continuous monitoring of physiological parameters offers a potential solution to assess personal healthcare. Identifying outliers or anomalies in heart rates and other features can help identify patterns that can play a significant role in understanding the underlying cause of disease states. Since anomalies are present within the vast amount of data generated by wearable device sensors, identifying anomalies requires accurate automated techniques. Given the clinical significance of anomalies and their impact on diagnosis and treatment, a wide range of detection methods have been proposed to detect anomalies. Much of what is reported herein is based on previously published literature. Clinical studies employing wearable devices are also increasing. In this article, we review the nature of the wearables-associated data and the downstream processing methods for detecting anomalies. In addition, we also review supervised and un-supervised techniques as well as semi-supervised methods that overcome the challenges of missing and un-annotated healthcare data.

## 1. Introduction

The assistance of wearable technologies in monitoring healthcare is revolutionizing the medical field. The emergence of wearable devices has allowed real time monitoring of vital signs, including the heart rate, number of steps taken and other parameters, such as calories and elevation [1]. These devices enable the continuous and longitudinal monitoring of the above-mentioned physiological parameters. The advantage of such a system is that it can be used anywhere and at any time.

Given the clinical significance of wearable devices and the associated physiological parameters that are measured, wearable devices likely can play a role in reducing health check-up costs and unwanted burden on the already overloaded healthcare programs around the world. The role of wearables in the future of precision health is currently being developed on a large scale [2]. To obtain an overview of the number of studies in the field of wearables, a keyword-based search was performed. The term “wearable sensors healthcare monitoring” was searched using the Google Scholar database (accessed 18 October 2021). We observed that the number of wearables-related studies has significantly increased within the last two decades (Figure S1).

The increase in wearables-associated studies published in the last few decades indicates a growing interest in wearable applications and efforts in advancing the data analysis associated with this field. The growing trend observed here also indicates the growth of the wearables market with several companies now developing biosensors for monitoring physiological parameters.

Several smart wearable devices and/or applications ranging from smart clothes, skin devices and other gadgets have been developed [3]. Popular clinical and research-grade wearable sensor technologies are being manufactured by Ava Science, Abbot, Zoll and Medtronic, among others. These manufacturers have also been approved by the United States (US) Food and Drug Administration (FDA). Among them is also Fitbit^®^, which has the largest user base along with Jawbone^®^. These devices have shown good performance in terms of accuracy of user physiological parameters, which has led to them being employed for various scientific studies.

Apart from these devices, the Apple^®^ Watch 2, Samsung Gear S3^®^, Xiaomi Mi^®^ and Huawei Talk Band B2^®^ are other wearable devices that provide health care monitoring [4,5]. In addition to these established names in the wearables industries, there are several manufacturers that assemble devices, including Welch Allyn, Scanadu Scout and iHealth-finger. These latter firms have even been utilized in clinical studies [6]. Deemed a medical revolution, these devices provide continuous longitudinal monitoring.

Data processing of physiological parameters, such as heart rate, blood pressure and body temperature, recorded by the wearable devices, can provide a clinical yield that may play a role in assessing patient health. The continuous availability of the aforementioned features within well-defined time and date frames increases the availability of multiple unique patient data points individually as well as for larger population groups. In addition, such continuous monitoring may foster more efficient and reliable diagnoses.

Progress in the field of wearable technologies has also facilitated the development of algorithms for automated health event prediction along with modes for prevention and focused clinical intervention [7]. Extensive reviews on remote sensing of patient health and sensor development in the past have been presented, and such reviews continue to be published today [8,9,10,11]. Literature reviews and meta-analysis have also focused on wearable-based interventions, specifically on their role in enabling a healthier lifestyle [12].

In this review article, we address the process of detecting clinically relevant features extracted from wearable sensors and the associated data. As part of this process, a major task is to identify relevant data points or instances from raw wearable outputs which are indicative of patient health. For example, the heart rate is a vital physiological parameter, and abnormal heart rates that span a period of time can be translated into indicators of various diseases by utilizing mathematical models, which are elaborated from Section 3 onwards.

These abnormal data points are called anomalies, and this review is primarily on understanding and reviewing algorithms that are capable of detecting anomalies in addition to decision-based systems that can handle the constantly evolving personalized data [13]. Even though methods exist for anomaly detection, there are still some challenges in the current literature with respect to anomaly detection. The first hurdle in anomaly detection is in evaluating the anomalies and distinguishing them between true and false positives.

Secondly, various statistical and machine-learning-based techniques are employed in anomaly detection based on the field of application, such as bank fraud, malware detection and healthcare. However, the prediction of anomalies in each field is based on trends and signatures which are unique to that field [14]. In the field of healthcare, application of the existing anomaly detection methods would require significant re-structuring and unique assumptions [15]. In this article, we provide an overview of anomaly detection, data types, imputation strategies and the prospects of the field.

## 2. Overview of Anomaly Detection

This section details the data types and strategies involved in analyzing the wearable data for detecting anomalies. The process of anomaly detection involves detecting patterns in heart rate and steps among other parameters that are significantly different from the remaining data. Anomalies in heart rate typically translate to significant and often actionable information.

In addition to the enormous value of heart data, other physiological data that can be collected from wearables include steps, blood pressure, the respiration rate, SpO2 levels, electrocardiogram, calories and skin temperature [16,17]. Additionally, various studies have combined capnography, stroke volume, pain, level of consciousness and urine output to accurately determine the associated physiological changes in patients. Recent studies that will be discussed further have shown that there are few traditional vital parameters that are crucial and can accurately evaluate human health.

Heart rate is considered as the standard vital sign indicating changes in cycles of the heart. Recent studies have shown an increase in the usage of this primary attribute to infer various cardiac pathologies [18,19,20]. Evidence is mounting in support of heart rate data to assess cardiovascular disease and its prevention [21]. A high resting heart rate correlates with an increased risk of coronary artery disease (CAD) [22]. Even in healthy people, heart rate monitoring gives an insight into normal cardiac physiology.

**Figure 1:**
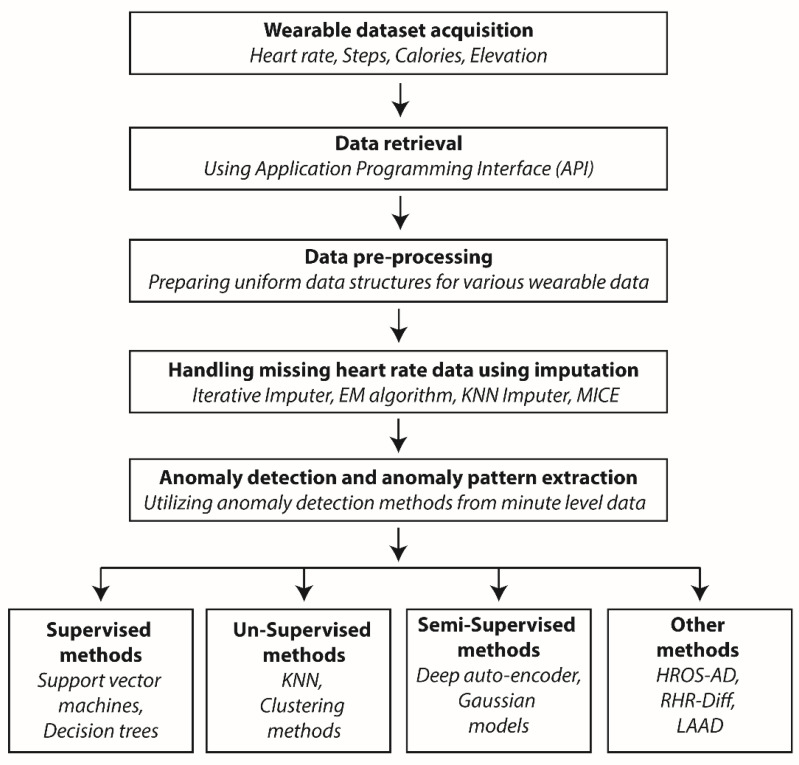
Flow chart describing a general anomaly detection workflow.

Monitoring healthy individuals is necessary as anomaly detection techniques employ supervised, un-supervised and semi-supervised algorithms, all of which requires continuous temporal data for analysis. Using anomaly detection, the identification of unusual patterns can sometimes be false positives and will not have any medical relevance; hence, the results obtained by anomaly detection methods should always be cross-checked with the electronic health record (EHR) data of the user. A general overview of anomaly detection is described below (Figure 1).

*The process starts with acquiring data from patient wearable devices, which includes the heart rate and steps. Various API interfaces are available to download this information, and these use secure gateways to retrieve it. The raw data are then pre-processed to ensure all the parameters have a uniform data structure based on timestamps. Data pre-processing is followed by missing data imputation by utilizing various algorithms, e.g., the Expectation-Maximization (EM) algorithm. The processed data are then subjected to various statistical and machine-learning algorithms for anomaly detection.*

*Anomaly detection can be carried out using several methods as elaborated at the end of the workflow. Some of the methods comprise hybrid methods from statistics, machine learning and data analysis, for example, HROS-AD, RHR-diff and LAAD. The detected anomalies are used to predict and infer clinically relevant information for the user wearing the wearable device. Abbreviations: EM—Expectation Maximization; KNN—K-nearest neighbor; HROS-AD—Heart rate over steps anomaly detection; RHR-Diff—Resting Heart Rate difference; LAAD—Long Short-Term Memory Network-based autoencoder; and MICE—Multivariate imputation by chained equations.*

### 2.1. Noise and Outliers

Anomaly detection is strictly distinct to noise in the data. As the word suggests, noise is a phenomenon that is of no interest to data analysis and must be removed before anomaly detection is carried out. Considering a subjective judgement, the designated deviation for a point to be called an outlier in real applications is a difficult task. The anomalies can be embedded in a huge amount of noise, and it is noteworthy that even an outlier should be considered an abnormality/noise. The problem with noise in data is far worse for electronic health records since very little information is available on the patient’s whereabouts, which is required to associate the physiological parameters.

**Figure 2:**
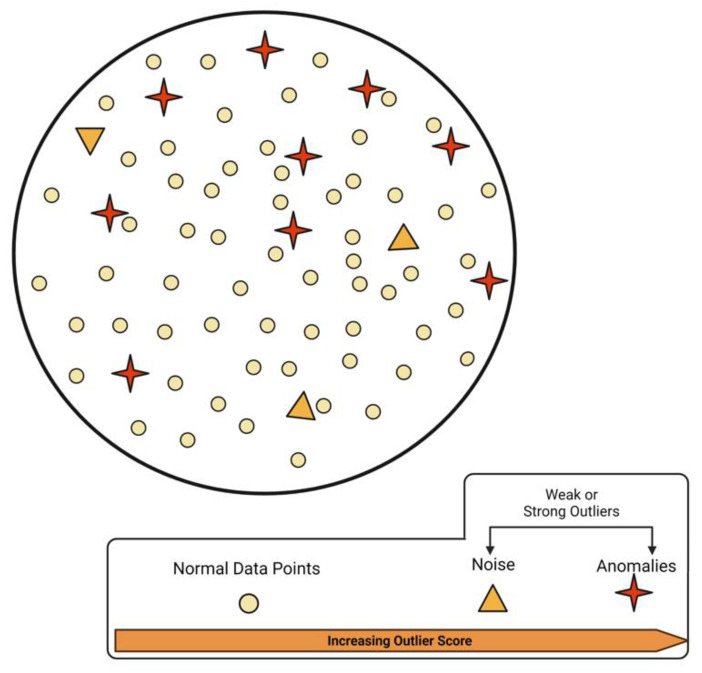
A schematic of normal, anomaly and noise data mixed in a wearable dataset.

Under such conditions, noise is considered a weak outlier, and its detection algorithms use quantifiable methods, such as the nearest neighbor algorithms [23]. Since wearable data comprises continuous time series data points, careful evaluation is required for the distinction between normal data, anomalies and noise. However, some studies suggest that even the outliers may contain valuable information [24]. Raw data comprised of multiple components is illustrated in Figure 2.

*These data instances require meticulous differentiation for further processing as discussed by Aggarwal (2017)* [23]. *In wearables-associated data, the distinction between normal and other data types is primarily performed using machine-learning-based techniques.*

Various statistical tests along with proximity models provide a good approximation for differentiating normal data points from the rest [24]. Furthermore, the knowledge of outlier detection also requires understanding of the different machine-learning-based categories. Studies in anomaly detection problems in the area of finance, climate and internet applications have broadly used supervised, un-supervised and semi-supervised machine-learning approaches [25,26].

Semi-supervised methods have been successful and applications in the above fields have displayed methods, including the Mahalanobis distance [27], Cook’s distance [28], Tukey’s method [29], Z-score [30] and K-means [31] and K-medoids [24].

However, there are a few challenges to be addressed before considering anomaly detection from wearables-associated data. First, defining a region of anomaly is often in the boundaries of normal patterns. Under such situations, normal physiological behaviors can often be masked as anomalous and vice versa. Secondly, in the case of heart rate data, normal behavior might evolve, which can be otherwise represented in the absence of adequate knowledge of underlying physiological changes. Another important challenge is the unavailability of labelled data, which is usually used for training or validation processes. Under such conditions, appropriate modifications in the above-mentioned algorithms, as elaborated in Section 4 of this article, are required.

### 2.2. Data Types

The data collected from wearables can be an object, point or vector among others. The data can have attributes that can be binary, continuous or categorical. These data can have a relationship with each other in the form of sequence data, spatial data and graphical data. The anomaly points from these data can be divided into point anomalies and contextual anomalies. While point anomalies are those instances from the data that are anomalous or those that lie outside the boundary of the normal regions, the latter categorizes points based on a specific context. They may be deemed as an outlier in one instance while being normal in another [32].

The contextual anomalies are dependent on the structure of the data set and anomalies detected from time series data are a prominent example. Another group, called collective anomalies, is the occurrence of points that are not anomalous by themselves but occur as a collection of points as seen in cases where the data instances are related [33]. In a particular study, Chandola et al. (2009) introduced an example of human electrocardiogram output in which a region of low value existed for a large time period. In such cases, this abnormality was not characterized as an anomaly.

Labels provide additional information about each instance in the wearables data. A significant feature of advanced wearable devices is the availability of several features that can enhance the value of each point. Along with heart rate, information on the number of steps, calorie consumption and temperature can aid the anomaly detection methods. Retrieving these labels, however, poses a challenge [34]. The best practices in anomaly detection include associating wearable devices data with patient health records, which can annotate each instance of heart rate, steps, calorie consumption etc., into medically relevant information clusters [35]. However, obtaining such data from health care management systems depends heavily on accurate measurements and a thorough knowledge of the underlying mechanisms.

### 2.3. Data Pre-Processing

Wearable devices, based on their capability, can detect various physiological measurements from their users. These include heart rate, steps, calories burned, elevation and various activity summary data at the seconds and/or minute levels. Several wearable manufacturers have also made available dedicated servers for storing the user information, e.g., Fitbit^®^ stores user data in their servers. The raw data can be accessed via an application programming interface (API) and user authentication [36]. Once downloaded, these files can be converted into programmable formats for further processing.

The most popular among these are comma-separated values (CSV) files due to their pre-dominance in the data landscape [37]. The wearable data, which includes all the physiological properties described above, can be downloaded for each day separately. These intraday data are typically processed in order to achieve uniform date–time stamps. The anomaly detection pipelines have different requirements based on their usage. Although, all of them need the heart rate and the corresponding time frames, which has to be built accordingly.

Machine-learning-based methods generally require one file with all the physiological annotations that could be extracted from the device. These methods are mostly unsupervised and are capable of predicting accurate anomalies from user data. The LAAD framework has been particularly successful in this respect, and this, along with other existing methods, will be elaborated in greater detail in Section 3. A major challenge in the pre-processing step is filling and/or imputing the missing data [38], which is detailed in the following section.

### 2.4. Missing Data and Data Imputation

The physiological data from daily activities gathered from wearable devices at a highly detailed level can yield accurate clinical information. However, retrieving continuous wearables data is a difficult task due to inherent “missing data” challenges. There can be a number of reasons why data is missing from a wearable device.

For example, instrument malfunctions as well as inconsistencies in extracting all of the data due to abrupt wearing behaviors constitute a few of the reasons [39]. Imputing missing data may be suspect and draw great attention to the validity in time-series data analysis. Missing data can be categorized into three types based on the likelihood of being missing; missing completely at random (MCAR), missing at random (MAR) and missing not at random (MNAR). In MCAR, no difference exists between data sets with missing data and those with no missing data.

Missing data caused due to power loss in the wearable device is a loss of information, which is due to random chance. In the case of MAR, there is a pattern where the data would be missing based on the time period when the battery is still active. If the data were missing only on that specific time of day, it would be considered missing at random. It is assumed that, in the case of MAR, the missing data can be evaluated from the remaining values, i.e., we still have the information about the instance of the missing data. Here too, the missingness depends on the data we observe.

Here, the probability of the missing data is the same within certain specified instances. In both MAR and MCAR, we have a good chance of recovering the missing data. However in MNAR, the source of missing data is known, and yet, a mechanism to effectively retrieve it does not exist [40]. Wearable devices could be removed because of various reasons and sometimes even purposely, which could lead to MNAR. When the data follow a MCAR or MAR distribution, the observed data are still representative of the population, unlike the MNAR. The chief principle of identifying the appropriate category of missing data is so that no one fills in missing data unnecessarily.

Omitting the missing data can directly lead to biased estimates. Hence, data imputation presents the alternative by estimation based on knowledge from data and predicting the values. The approaches to handle missing data are broadly distinguished into statistical imputation methods and imputation mechanisms that are integrated with machine-learning algorithms. One of the most basic methods of missing data imputation is mean value imputation.

In this method, the empty instances are filled using the mean values from the available observed data. However, this method is unable to preserve the relationship between variables. Since the imputation in this case are estimates, there will be associated errors, which will lead to committing type I errors, in which case, the null hypothesis will be rejected although accurate. Although there are several methods for handling missing data, two of the widely used approaches include the maximum likelihood (ML) and multiple imputation (MI) methods.

The maximum likelihood method is considered to be more efficient due to multiple reasons, including the minimum sampling variance, consistency in the results produced and one uniform model. On the other hand, the multiple imputation method requires several decisions and imputation models, etc. Hot deck imputation is a simple and effective method of imputing values from the observed set of a similar unit, i.e., imputing values using values from the same set. This method is not as developed as the other methods, and its applications may have challenges which are yet to be studied in detail.

The multivariate imputation by chained equations (MICE) method imputes data using assumptions like MAR and MCAR [41]. The iterative series of the prediction models employs multiple variables to impute the missing variable making it highly robust. This is particularly useful for imputing values belonging to variables with good correlation. Bootstrapping along with multiple imputation using expectation maximization (EMB) is a robust method. This method is known to be effective for up to 30% missing data and is far superior to single imputation methods which do not consider the uncertainty of the predicted missing value as they are obtained using the mean, median or mode as opposed to the machine-learning-based methods, such as MICE. A brief comparison of these methods is presented in Table 1.

## 3. Basic Categorization of Anomaly Detection

The wearable data has two main features: a timestamp and continuity of data points. Each variable must have a time dimension and data points that are continuous within a certain period of time. Repeated measurements over time, otherwise referred to as time-series data are the primary form of data structure retrieved from wearables [48].

The physiological measurements, such as heart rate, steps etc., that are collected at regular intervals over time allows the identification of trends in patient health and aids in health monitoring. Time series data can be further classified into two types: univariate and multivariate. Univariate time series have scalar values, while the latter consist of multidimensional values or vectors. Wearable associated data are often multivariate time series as these consist of more than one-time dependent variable [49]. These variables are also dependent on each other.

In addition to the type of data, it is also important to understand the different types of outliers that are often discussed within the context of anomaly detection in multivariate data. Point outliers are abnormal points at a specific instance of time. The abnormality could be judged based on its relationship with the subsequent values, which is detailed in the next section. The other type of outliers is categorized as subsequence outliers. These are a collection of consecutive outlier points that can affect multiple variables in the dataset [23,50]. Detecting these outliers/anomalies from wearables-associated data would require a combination of several existing methods. These existing methods are benchmark anomaly detection techniques, which have been previously employed in fields, such as bank frauds, data networking, spam detection, insurance and other fields.

The methods employed to identify anomalies in these areas are now observed to have extended applications [25,51]. A certain degree of similarity in the data types of the above-mentioned fields with that of wearable data for example, time instances, longitudinal data and the existence of baseline features, among others, have pushed researchers in the field of anomaly detection to apply the existing algorithms for identifying health-related anomalies in the wearable data. As previously stated, anomaly detection for wearables-associated data can be broadly divided into supervised, semi-supervised and unsupervised methods.

### 3.1. Supervised Anomaly Detection

In this framework, the dataset is composed of labels for training and test data. The training data can be used to create a classification model that will demarcate the normal versus the anomalous instances. The general principles behind these methods are often independent of the spatial or temporal data in use [52]. The chief goal of supervised anomaly detection is to support the learning method with application specific information. In the case of wearables, the goal is to identify physiological data points that give information about the hidden abnormalities in the users’ data. Such relevant markers are however very rare to extract since extracting continuous biologically relevant information for long time periods and without missing data is difficult. As a consequence, the creation of a supervised learning model is very difficult [53].

A clear distinction between normal and abnormal data is required for supervision and is also referred to as training. Acquiring label information on these data points is of the utmost importance. Supervised anomaly detection methods completely ignore unlabeled data in the training step. The dependence of supervised methods on labeled data includes active learning from the extensive knowledge available for the outlier candidates [54]. It is evident that supervised methods would provide a better accuracy of detection by virtue of the additional knowledge.

Some of the popular methods include supervised neural networks, support vector machines (SVM) and decision trees. Support vector machines has been extended to various non-standard scenarios as in the case of anomaly detection from wearables [55]. Training data are used by the one-class SVMs, which then model the density distribution based on it. This is later used to differentiate between normal and abnormal data instances. Another popular method is the application of decision trees. Decision trees create models that classify anomalies based on a decision rule. This rule is dependent on the training data.

The classification of anomalies depends on the model class that is evaluated from the training data. The classification of data points will depend on all the nodes of the decision tree and its corresponding value derived from the decision rule. However, decision trees can produce over-complex trees [56]. Depending on the type of device, healthcare data from wearables can have several annotations, which can be useful for increasing the accuracy of machine-learning-based predictions. Several new approaches have been designed that addresses the current drawbacks, and these are detailed in Section 4.

### 3.2. Unsupervised Anomaly Detection

Contrary to supervised methods, this framework does not require training data. These algorithms are based on two assumptions for the data under consideration. The first assumption is that the number of normal instances far outnumber the anomalous ones. The second assumption views anomalies as a different qualitative instance compared to the normal [57,58]. Data groups appearing frequently can be assumed normal. Based on these assumptions, unsupervised anomaly detection methods can be broadly categorized into clustering-based methods, statistical methods and also nearest neighbor-based methods, the latter being frequently used for anomaly detection in this category [59].

k-Nearest neighbor (KNN) in the field of anomaly detection is a classification method based on user-defined threshold values. Even though the KNN method is used in supervised anomaly detection, it is also applicable in unsupervised anomaly detection. The fundamental idea behind identifying an anomaly is usually its tendency to stay farther away from a cluster of similar observation. In the absence of learning, a threshold value has to be determined, which will determine the anomaly [60]. However, a major drawback is that it identifies only global anomalies or point anomalies. In wearables data, local anomalies are equally important, as anomalies at an instance of time can have a significant impact on the nearby values. In any medical condition, the instances before and after an anomaly are particularly relevant and important.

Some of the widely used unsupervised anomaly detection methods also include the clustering technique [60,61] unsupervised neural networks [62] and K-means-based clustering method [63]. Neural networks are particularly important for anomaly detection in multivariate data, which is primarily the case with wearables-associated data. These can correlate different variables of the time series. A major drawback of supervised methods, such as SVM and KNN is their low effectiveness against multivariate data.

Several modifications on neural network-based methods, such as deep auto encoding [51], Gaussian mixture model [64], convolutional long short-term memory networks [65], multi-scale convolutional recurrent encoder-decoder [66] are amongst the latest improvised methods to overcome such issues. Several algorithms have been proposed for this unsupervised anomaly detection but to identify the proper subset for the anomaly detection task is considered difficult. With respect to the unsupervised anomaly detection method, deep learning allows extraction of the features from the data. There are, however, a few limitations to this approach.

It is difficult to obtain such huge volumes for training purpose. A large section of these data is unstructured and requires various pre-processing steps, which may vary based on their sources [67]. There also exists an innate variability amongst anomalies themselves. There are various types and subtypes of anomalies, with 3 broad, 9 basic and 63 subtypes, which have been previously studied. These should be understood in the analysis of healthcare associated data as further research work is done in anomaly detection [68].

### 3.3. Semi-Supervised Anomaly Detection

The semi-supervised detection method is considered to be better than supervised methods. Often due to incomplete information in data (mostly outlier labels and the variance in outlier (randomness of values)), semi-supervised techniques are used to check the outlier property of a data point. This class of anomaly detection is involved when a large section of the data is unlabeled and the dimensions of data are high, which is especially the case with clinical observation data [69].

Traditional unsupervised methods can work on unlabeled data; however, the unavailability of large-scale continuous data can be a major limitation. This is another reason behind employing semi-supervised methods, which can work with missing data at the cost of few labeled instances.

The aim of such detection methods is to build a normal class model, and the anomaly can be detected based on the deviation of its corresponding instances [70]. Widely used methods in this category of anomaly detection methods include one-class SVMs, deep auto-encoders and Gaussian models [64,71,72,73]. Deep semi-supervised anomaly detection was also introduced recently, which not only learns from labelled normal data but also labelled anomalies [74].

This also, like the supervised and un-supervised method, has to be applied under definite assumptions with a relative consideration towards the limitations. However, a combination of statistical inference along with machine learning methods is mostly used for anomaly detection in a disease framework. These methods are thus tailored to address the availability of continuous data, the type of devices and the disease conditions.

## 4. Applications of Anomaly Detection Methods on Wearables Associated Data

There are several wearables-associated studies with clinical implications. In this section, we elaborate in detail some of the most relevant studies. Li et al. (2017), in their study, demonstrated that wearables have the potential to monitor activity along with physiology. In the study, they combined the sensor data with frequent medical measurements to make predictions on the onset of Lyme disease and inflammation. Through the study, they also observed a distinction between the insulin-sensitive and insulin-resistant subjects.

Various devices were employed that measured the heart rate, SpO2 and temperature along with other activity-related parameters for a prolonged span of two years. A baseline was established for the data during sleep and non-sleep instances, and based on this, a Z-transformation was employed to scale and standardize the time instances. The activity specific data was then monitored and compared with the overall period to reveal the outliers [6].

In a study by Mishra et al. (2020), heart rate and steps data were used to detect COVID-19. They analyzed physiological and activity data from over 5200 participants, which included 32 individuals diagnosed with COVID-19 infections. The study showed elevated resting heart rates relative to the individual’s baseline. With the missing values imputed as zeros, two algorithms resting heart rate difference (RHR-diff) and heart rate over steps anomaly detection (HROS-AD) were developed. The first algorithm was based on standardizing the resting heart rate over a fixed time frame to observe baseline residuals.

The time interval was detected as anomalies based on scan statistic method [75]. In HROS-AD, heart rate and steps data were combined in a machine-learning-based elliptic envelope approach. The data was assumed to have a Gaussian distribution and the method identified both univariate and multivariate outliers based on the distance of each HROS point from the overall mean. These points are regarded as contaminants that are too extreme for the assumed Gaussian distribution of data [76].

In a similar study, Bogu et al. (2021) were able to detect an abnormal resting heart rate during the COVID-19 disease state. The resting heart rate was identified using corresponding step count values. A deep-learning approach based on a Long Short-Term Memory Network-based autoencoder (LAAD) was employed in this study. Data labelling (infectious, non-infectious and recovery periods) was based on a literature review. The abnormal resting heart rate (RHR) was estimated using its distance from baseline before generating the training set.

To prevent overfitting in the deep learning model, different data augmentation techniques were employed, which increased the size of training data. RHR indicative of COVID-19 infection was detected in 14 individuals [77]. The aforementioned studies emphasized the importance of characterizing an individual’s baseline. Irrespective of the evaluation models applied, setting a baseline can significantly improve the performance of anomaly detection.

Another study performed by Zhu et al., on COVID-19 prediction by utilizing heart rate and sleep data collected from wearables devices, employed a neural network prediction-based method. This study used categorical features, such as the holiday activity the person was engaged in along with information on the current season and weather patterns in addition to the wearable data. This information was combined with historically detected anomaly rates to construct the input for anomaly detection [78]. To accurately determine the anomaly points, it is important to identify the source of those points.

Various physiological ailments can point towards the anomalies; hence, a wider range of categorical features was proposed in this study. Apart from COVID-19, heart related ailments can also be monitored using wearables. Wang et al. (2021) studied a total of 16,320 atrial fibrillation (AF) patients and established that wearable use has a positive effect on health care [79]. The detection of AF was successfully performed by Lown et al. (2020).

In this case, an online library of time interval between two consecutive R-waves from an electrocardiogram was used, and a correlation of RR interval changes was studied with respect to previous changes, which represented the heart rate variability. These changes were represented using a Lorenz plot. A Gaussian support vector machine was employed, which correctly identified AF with 100% accuracy [80].

These studies exemplified wearable technology and its application in real time disease state monitoring. Hybrid algorithms profiting from the available statistical and machine learning frameworks are expected to grow in the near future. Some examples of such research works are presented below (Table 2).

## 5. Prospects

### 5.1. Handling and Transparency of Wearables Associated Data

Extracting clinically relevant outputs from wearables-associated data has several key requirements. The anomaly detection algorithms discussed above have all greatly benefited from the availability of large wearable datasets; hence, the development of data repositories should go hand in hand. A slow but steady shift from hospital-based care to patient-centric care is being observed worldwide. This, along with an increasing popularity of wearables, is slowly leading toward a data surge [87,88]. Tracking the progression of any disease state demands uninterrupted wearables-associated data points, which is beyond the scope of hospital care.

Such longitudinal data collected over large time periods on a daily basis can amount to data accumulation. The analytical usage of this data, as it has been discussed throughout this current review article, depends on approaches to access and distribute the data. The development of wearables-associated software directed at both accurate health monitoring and state of the art data collection, analysis and visualization is of the utmost importance [89]. Both standalone and hybrid methods for anomaly detection require accurate interpretations that can be correlated with the user activity and their daily experience.

Generating recommendations includes decision making by the users and goal synchronization, both of which may require platforms in addition to mobile applications. Existing recommendations regarding the correlation of various variables from the device should be put to the test. For example, exercise and sleeping are independent variables, which, when correlated can conceal the underlying physiological anomaly. Additionally, statistical significance on relationships should leave enough room for practical significance, which arises with the increase in data.

Anomaly detection methods also needs gold-standard data against which to compare the generated results. Databases, like the UK Biobank [90], and other similar libraries with cohort studies can be very useful in comparing standalone wearables-associated studies. Another important area is the transparency of algorithms designed to compute steps or sleep data, which should be encouraged. The limitations of the large-scale data generated from wearables need to be addressed more often [89].

### 5.2. Application of Wearables in Healthcare

The impact of COVID-19 has renewed everyone’s interest towards novel healthcare solutions. Wearables-technology and its adoption is significantly increasing in awareness among the public. Wearables are already being integrated into clinical practices. The vital signs, such as heart rate, temperature, blood pressure and blood oxygen saturation, measured by the wearable devices are carefully being used for clinical applications. The extraction of useful health-related markers is slowly making its way into mobile health-related interventions [90].

Intensive care units (ICU) have reported many benefits by employing wearables in making precise medical decisions. Heart rate and sleep measurements have helped manage post-ICU care. Patients recovering from major surgeries and associated stress can benefit from a continuous monitoring of their physiological changes. Heart rate and sleep are often associated with pain levels and underlying recovery.

Wearables-derived data employed with machine-learning algorithms can, therefore, provide accurate patient monitoring. A wide array of disorders is now being brought under the investigation paradigm of wearables. Metabolic disorders are marked by high blood pressure, blood sugar and abnormal cholesterol, among other conditions. With advancements in non-invasive monitoring of such conditions, the management and control of disease becomes easier [7,42]. Efforts are being made to monitor and diagnose hypertension by measuring blood pressure longitudinally over time. Blood pressure monitoring can be helpful for many associated primary conditions [91,92].

Additionally, sleep disorders, mental health, maternal and neo-natal care require active tracking; they can benefit from wearable technologies. Monitoring patient fitness has allowed for a better understanding of the before and after disease states. Such initiatives have afforded significant observations in various disease models. In case of cancer treatments, analyzing biometric data from wearables can be used to predict pain levels, stress etc. Similarly, activity and sleep data have been collected and employed to predict migraine attacks.

In this particular clinical trial, machine learning was employed on a dataset comprised of physiological parameters along with environment variables to predict the likelihood of a migraine attack [93]. Such studies have been noted from the ClinicalTrails.gov database. There are 82 studies that have wearable devices associated in their clinical trial process, twenty (20) of them have been completed thus far, and 36 are in the recruiting state. Other studies (14) have been initiated pending recruitment, while the remaining studies are either suspended or the status is unknown. A detailed account can be found in Table S1.

Among the current on-going clinical trials, the wearables-associated interventions have been particularly successful in managing lifestyle diseases. Metabolic disorders and their risk in worsening cardiovascular disease state is an active field of study in the application of wearables. Based on evaluating the disease conditions in the ongoing trails, there are 23 clinical trials utilizing wearables to understand and/or monitor lifestyle disease states. Hypertension requires a longitudinal measure of heart-rate variability, and wearable monitoring devices are an affordable and non-invasive means of monitoring the heart rate [94].

Monitoring energy expenditure in cardiac patients can be crucial in their rehabilitation. A Fitbit wearable-based clinical trial was able to assess the improvement in the physical activity of patients with chronic heart failure and coronary artery disease. Similarly, in demonstrating the effectiveness of a fitness tracker for children with deep vein thrombosis, a research team employed Fitbit devices to evaluate the physical activity in patients [95]. Several such studies are proving that wearables are robust healthcare monitoring devices.

### 5.3. Impact of Wearables on Managing Healthcare

Steady improvements in algorithms for anomaly detection are being made. Simultaneously, large datasets have been made available to improve the predictability. Wearables and the data analysis are thus connected to certain key areas, such as the cloud and data security. The internet connection, in particular Wi-Fi, helps in communication between wearable devices with hand-held devices, such as smart phones and the cloud.

The development of a dedicated cloud infrastructure for storage and analyzing data can enhance wearables usage in healthcare. The security of these devices is another aspect to be considered. A systematically regulated online cloud infrastructure along with device-to-device connectivity is necessary in digital healthcare framework [96]. These factors can eventually help in the application of wearables-associated big-data and accurate anomaly detection for studying disease pathogenesis.

Smartphones and other hand-held devices are typically used for obtaining and transmitting information collected from the wearables. An integrated system would be useful in managing disease states. Carefully detected anomalies with medical relevance can be translated into knowledge which can then be instrumental in handling a global health crisis. The COVID-19 pandemic has certainly shifted the thrust towards the need for a more digital or telemedicine-based healthcare system. The pandemic saw a rise in tele-consultations.

Under such situations, wearable devices can provide health parameters, which are otherwise collected during patient’s clinical visits. The primary health data collected in this way is already being used to assess chronic diseases. Tracking heart rate and other primary parameters has proved helpful in the past to develop models for the spread of influenza [97]. Such physiological tracking studies have been initiated by The Scripps Research Translational Institute along with Fitbit^®^, Apple^®^ Watch and Garmin^®^ devices [98].

Another study conducted by TemPredict at University of California, San Francisco (UCSF) utilizing the Oura ring also made efforts in tracking physiological data from users [99]. The Internet of Things (IoT) can integrate and store data and carry out smart identifications along with the tracing of patient data and exchange of knowledge. The entire infrastructure is a potential tool for doctors to handle crisis situations.

## 6. Conclusions

Wearables-associated data are increasingly becoming popular in clinical setups. Such data are extremely valuable to predict disease biomarkers. The application of anomaly detection methods in the rapidly growing healthcare field can aid clinical practices by detecting inconsistencies and undetected physiological parameters. The machine-learning approaches discussed in the review are being employed to not only detect outliers but also identify novel data points, which are far from otherwise seemingly normal physiological values.

These methods also address the drawbacks of un-annotated wearables data by employing semi-supervised methods, such as one-class support vector machines and deep auto-encoders, which employ training based on both labelled and unlabeled data resulting in accurate user data analysis. Overall, applying anomaly detection on the real-time data produced by wearable devices can reveal valuable clinical information in terms of disease diagnosis, prediction, treatment and rehabilitation.

## Figures and Tables

**Table 1:** Data imputation methods.

Methods	Definition	Accuracy	References
Mean value imputation (MVI)	The values are filled using calculating the mean for a missing value	Biased	[42]
Maximum Likelihood (ML)	A likelihood function is evaluated and then sum or integrate over the missing data	Unbiased parameter estimation	[43]
Hot Deck Imputation	A data matrix for all instances created is chosen as a source for missing values	Replication of values may cause bias	[44]
Multiple Imputation (MI)	Starts by introducing random variation and generates several datasets with slightly different imputed values. Statistical analysis on each to find the optimal one	Comparable to ML	[45]
Multivariate Imputation by Chained Equations (MICE)	The method first identifies an imputation model for each column followed by random draws from the observable data	Comparable to ML	[46]
Expectation–Maximization with Bootstrapping (EMB)	Initially the likelihood function is evaluated using model parameters. Next, with the updated parameters, the likelihood function is maximized, and the parameters are updated to return a new distribution	Comparable to ML	[47]

**Table 2:** Wearables-associated studies with clinical implications.

Disease under Study	Wearables Used	Method Applied	Major Finding	References
COVID-19	Huami wearable devices	Anomaly detection algorithm, neural network prediction modelling methodology	Prediction model with potential to alert COVID-19 outbreak in advance as a part of health surveillance system	[78]
Atrial Fibrillation (AFib)	Not mentioned	Not mentioned	Follow-up health care amongst those using wearables was higher indicating better disease management	[79]
Atrial Fibrillation (AFib)	Samsung Simband	Noise-resistant machine learning approach	The screening algorithm can enable large scale detection of undiagnosed AFib from noisy Photoplethysmogram (PPG) wearable sensor	[81]
Sleep/wake identification	Fitbit Alta; Fitbit Inc	Hidden Markov models	Accurate measurement of sleep/wake cycle and an effective personalized model	[82]
Monitor heart rate in real time during moderate exercise	Xiaomi Mi Band 2 and Garmin Vivosmart HR+	Not mentioned	Estimating accurate heart rate signals under physically strenuous activity	[83]
Prediction of Heart Failure Exacerbation	wearable sensor (Vital Connect, San Jose CA)	Machine learning analytics algorithm	Multivariate data from wearables accurately predicts the need for rehospitalization of patients with a heart failure risk	[84]
Atrial fibrillation (AF)	Amazfit Health Band 1S	Artificial intelligence (AI) algorithm	PPG sensor derived data along with AI can be an efficient way to detect AF	[85]
Distance walked or run, calorie consumption, quality of sleep and heart rate	Fitbit Charge 2 (Thought Technology LTD, Toronto, CANADA)	HR-derived algorithms	Accurate heart rate monitoring for fitness tracking using wearables compared to electrocardiograph has several significant differences, which needs to be studied	[86]

## Data Availability

Not applicable.

## Supplementary Materials

The following supporting information can be downloaded at: https://www.mdpi.com/article/10.3390/s22030756/s1, Figure S1: Comparison of number of research articles on wearables sensors and healthcare monitoring; Table S1: List of wearables-associated clinical trials.
