# Supplementary material for: Anomaly Detection Framework for Wearables Data: A Perspective Review on Data Concepts, Data Analysis Algorithms and Prospects
Source: Sensors (Basel). 2022 Jan 19;22(3):756. doi: 10.3390/s22030756 (PMC8840097; doi:10.3390/s22030756)
Supplement: Supplementary file 1 [file sensors-22-00756-s001.zip › Figure S1.pdf]

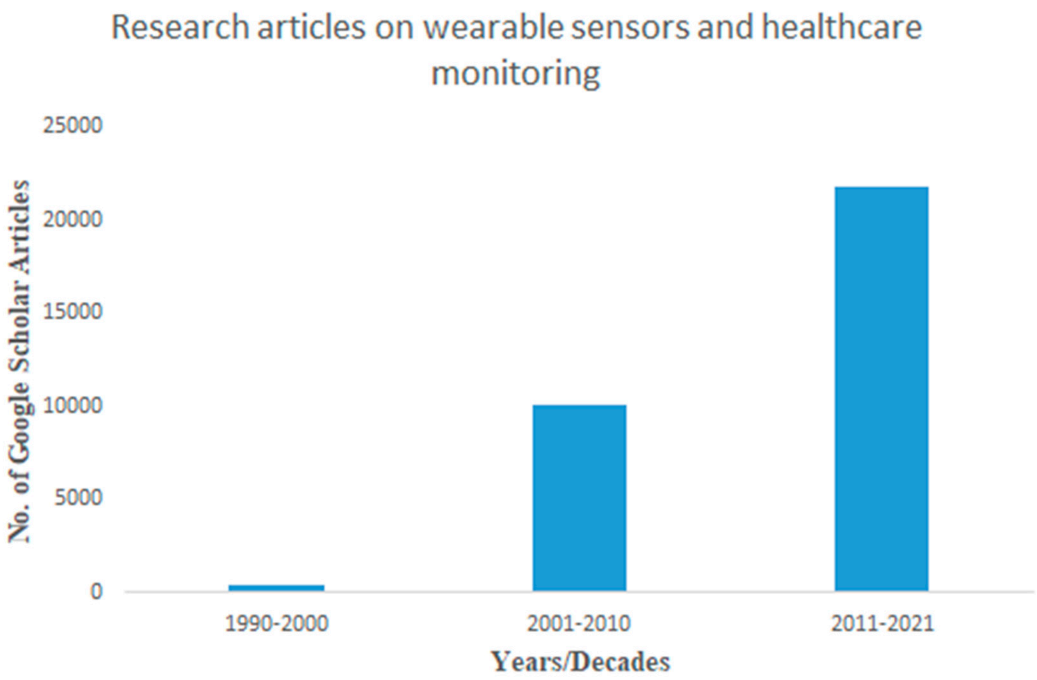

Figure S1. Comparison of number of research articles on wearables sensors and healthcare monitoring.
